# Digital biomarkers for early agitation detection in dementia: a scoping review of emerging wearable and smart technologies for personalized care

**DOI:** 10.3389/fneur.2026.1683517

**Published:** 2026-02-10

**Authors:** Alex Malioukis, R Sterling Snead, Julia Marczika, Radha Ambalavanan, Gideon Towett, Mercy Mbogori-Kairichi

**Affiliations:** The Self Research Institute, Broken Arrow, OK, United States

**Keywords:** agitation, biomarkers, dementia, early detection, personalized care, wearables

## Abstract

**Introduction:**

Agitation is a common and burdensome symptom in people with dementia, particularly when compounded by impaired communication, making early detection and effective management difficult. Wearable sensor technologies may offer a promising avenue for supporting real-time behavioral monitoring and personalized care in this context.

**Objective:**

This study aims to examine the current clinical and technological capabilities of wearable sensor systems for detecting and managing agitation in persons with dementia, to assess whether these technologies can effectively support personalized care. Additionally, it seeks to identify key challenges and opportunities in applying human-centered design principles and tailored interventions to improve outcomes for both patients and caregivers.

**Methods:**

We conducted a scoping literature review, registered on OSF^1^ and guided by PRISMA-ScR guidelines. Five databases—Google Scholar, Scopus, PubMed, PsycINFO, and ACM Digital Library—were searched for English-language peer-reviewed studies published between 2016 and early 2025. From an initial pool of 798 articles, a multi-phase screening process led to a final inclusion of 13 studies that met predefined criteria.

**Results:**

The reviewed studies demonstrated that wearable sensors, particularly those employing multimodal data and personalized machine learning models, enable reliable detection of agitation symptoms and support timely, tailored interventions. The concept of digital phenotyping emerged as a promising approach for capturing complex behavioral signatures, while user-centered design was identified as essential for adoption and long-term compliance.

**Discussion:**

The evidence identified in this scoping review indicates that wearable and multimodal sensor technologies may offer promising approaches for monitoring agitation in dementia, while acknowledging that the research remains in early stages. We recommend future research focus on large-scale, longitudinal validation and the expansion of these tools to other populations with communication challenges, such as individuals with autism spectrum disorder or traumatic brain injury.

**Systematic review registration:**

https://doi.org/10.17605/OSF.IO/DNHYM.

## 1 Introduction

Wearable sensors have evolved to demonstrate increased accuracy in measuring and monitoring various health aspects in humans. From tracking physical activities to monitoring sleep patterns and measuring vital signs like heart rate and blood pressure, these adaptable sensors have transformed into indispensable instruments. Empowering users with real-time data for informed well-being decisions, they play a crucial role in fostering comprehensive health and lifestyle management (1–5).

Beyond these applications, wearable sensors have also been explored as tools to assist individuals with illnesses affecting communication, in expressing their unmet needs digitally. In the past decade, significant research has focused on leveraging contemporary technological advancements to detect and better manage agitation events and distress in individuals exhibiting such symptoms due to specific neurological, neuropsychological, or neuropsychiatric disorders. Diseases such as dementia and autism spectrum disorder are at the forefront of this exploration, where the lack or inability to communicate often results in undetected agitation (6, 7). This agitation can gradually evolve into even more distressing symptoms, causing unsettling experiences for both the individual and their usual caregivers. In the specific context of this population, the imperative of early agitation detection becomes evident. Recognizing signs promptly plays a crucial role in initiating timely interventions, preventing situations from escalating, and ultimately contributing to an enhanced overall quality of life.

Both conditions significantly impact a large portion of the global population. Although patients with these disorders may not exhibit the same signs and/or experience similar challenges in expressing their own selves, agitation is one of the most common symptoms. Often, agitation is not the primary symptom but is linked to impaired communication and the struggle to convey internal intentions and emotions. For the scope of this paper, we shall concentrate on studies done on agitation in people with dementia (PwD). Dementia affects more than 55 million people worldwide as of 2020. The number is expected to nearly double every 20 years, to an estimated 78 million by 2030 and 139 million by 2050 (8).

For instance, nearly half of PwD experience agitation symptoms monthly, with 30% of those living at home affected. Approximately 80% of individuals experiencing medically meaningful symptoms retain an ongoing state of restlessness for a duration exceeding 6 months, whereas 20% of those initially devoid of symptoms encounter the development of such symptoms over a span of 2 years. The presence of agitation in these patients, exhibits a strong association with a diminished standard of living, giving rise to unfavorable encounters, obstructing participation in activities and relationships, and eliciting sentiments of helplessness and anger in those who provide care (9).

Defining agitation requires caution due to its subjective nature. Clinically, it is defined as “inappropriate verbal, vocal, or motor activity,” with the complexity of assessment compounded by both communication difficulties and the multifactorial nature of behaviors manifested (10). However, it is probably universally agreed that, irrespective of the definition of agitation, in the particular context of this population, the potential importance of early detection becomes clear. Early identification of signs can start appropriate interventions, prevent situations from worsening, and ultimately lead to a better quality of life. With the relatively recent development of multimodal sensing and advances in artificial intelligence, medicine has reached an epoch where the implementation of such technology for early detection does not only seem possible but also particularly appealing.

Despite the high prevalence and substantial consequences of agitation in dementia, current approaches to detection remain largely reactive. Agitation is typically recognized only after escalation, prompting behavioral or pharmacological interventions that may be less effective and potentially carry risks. Early agitation markers, such as subtle physiological changes, restlessness, or environmental interactions, often go unnoticed in daily life. This challenge is intensified by impaired communication in PwD, variability in caregiver interpretation, and the reliance on episodic, time-intensive clinical assessments that may miss real-world fluctuations.

Emerging technologies may offer a means to address these limitations. Wearable sensors, environmental monitoring systems, and artificial intelligence–based analytic methods can provide continuous tracking of physiological, behavioral, and contextual signals associated with agitation. These tools have the potential to detect early deviations from an individual’s baseline before overt behavioral escalation occurs. Personalized modeling approaches, in particular, align with the highly individual manifestation of agitation and may enhance both sensitivity and specificity.

Although technological advances have progressed rapidly, the research landscape remains fragmented across different study designs, sensor modalities, and analytic methods. A consolidated evidence base is needed to clarify the potential, limitations, and real-world feasibility of these emerging technologies.

In line with this rationale, this review aims to investigate not only the feasibility but also the effectiveness of wearable technologies in detecting early signs of agitation in PwD. Drawing from the emerging evidence on sensor accuracy, algorithmic development, and the need for patient-centered personalization, we propose the following research hypotheses: First, we hypothesize that wearable sensor systems may be capable of detecting early signs of agitation in PwD. This is based on the growing body of literature indicating that multimodal sensing can support real-time behavioral monitoring and signal the onset of distress even in non-communicative populations. Second, we hypothesize that personalized machine learning (ML) models can offer improved accuracy in agitation detection compared with generic approaches. Given the inter-individual variability in dementia symptomatology and response to stressors, this hypothesis underscores the importance of individual-level adaptation in technological systems to ensure clinically meaningful and ethically sound interventions.

These hypotheses serve as the guiding framework for this review, aiming to synthesize the current evidence while identifying key areas for future investigation and clinical translation. In addition to mapping technological capabilities, we also tried to examine the challenges associated with integrating them into the caregiving community while prioritizing patients’ rights. Additionally, we aimed to identify key gaps and limitations in the current landscape, with a continued emphasis on promoting a human-centered design in future developments. Central to the implementation of this study is the notion that interventions should ultimately simplify the lives of those we support, enhancing their overall well-being and quality of life.

## 2 Methodology

### 2.1 Search strategy

We performed a review of sensor technologies applied for the detection and management of agitation in PwD. The study was registered as a scoping review protocol on OSF; https://doi.org/10.17605/OSF.IO/DNHYM. We used the PRISMA-ScR guidelines as a general framework to guide our review (11) (see Supplementary File S1 for PRISMA-ScR checklist). In doing so, five scientific literature databases-namely Google Scholar, Scopus, PubMed, PsycINFO, and the ACM Digital Library-were electronically searched. The search focused on studies published from 2016 through February 2025. In order to have a wide search to retrieve related articles efficiently, we used a strong set of keywords that involved all the essential and supporting terms (see Table 1). The search for relevant articles was conducted from January 5 to February 9, 2025, and was limited to English-language publications only. Thematic and keyword search results were then manually screened for relevance to dementia and the utilization of technological solutions in detection strategies. To this end, each study was carefully examined in relation to the objectives of our review, with an emphasis on sensor-based monitoring technologies capable of detecting and responding to agitation and other behavioral symptoms associated with dementia.

**Table 1:** List of keywords and boolean search strings used in literature search.

Keywords	Boolean search strings
Dementia, agitation, psychomotor agitation, behavioral symptoms, monitoring, wearable devices, smartwatch, environmental sensors, multimodal sensors, sensor technologies, artificial intelligence, digital phenotyping, machine learning, multimodal sensing, personalized care, blood volume pulse, skin conductance, skin temperature, accelerometer, contextual factors, psychometrics, real-time monitoring, physiological data, sensor data integration, early detection, intervention strategies	(“dementia” OR “communication-impaired neurological disorders”) AND (“agitation” OR “psychomotor agitation” OR “behavioral symptoms” OR “restlessness” OR “disruptive behavior”) AND (“wearable devices” OR “smartwatches” OR “environmental sensors” OR “multimodal sensors” OR “sensor technologies” OR “physiological data” OR “sensor data integration”) AND (“artificial intelligence” OR “AI” OR “real-time monitoring” OR “early detection” OR “intervention strategies”)

### 2.2 Criteria

Inclusion Criteria: (1) Studies focusing on the utilization of sensor technologies (e.g., wearable sensors, actigraphy, environmental sensors, multimodal sensing, EEG, computer vision) for monitoring and managing agitation events specifically in individuals diagnosed with dementia. (2) Articles discussing the application of cutting-edge technologies such as artificial intelligence (AI) and ML within the context of dementia care and utilizing sensor data. (3) Peer-reviewed journal articles, conference papers, and review articles that present empirical findings, theoretical frameworks, or innovative methodologies related to sensor-based approaches in dementia care. (4) Publications in English, ensuring accessibility to a wider audience and facilitating comprehension and dissemination of findings within the scientific community.

Exclusion Criteria: (1) Studies unrelated to dementia or agitation behaviors. (2) Non-human studies. (3) Research lacking involvement with sensor-based monitoring technologies. (4) Non-peer-reviewed literature, including editorials, opinion pieces, and case reports. (5) Publications not in the English language.

### 2.3 Data synthesis

The authors have independently screened this article for inclusion and exclusion criteria as described above. Specifically, four reviewers (AM, RS, JM, and RA) independently assessed the titles, abstracts, and full texts of the retrieved studies against the predefined criteria. At each stage, disagreements were flagged and discussed during team meetings. A consensus-based reconciliation process was employed to resolve any discrepancies in inclusion decisions. This multi-reviewer approach was implemented to minimize bias and ensure consistency in study selection. We initially identified 798 articles from the keyword search from the identified databases above. By limiting our selection to only peer-reviewed journal articles in the English language for which full texts were available, we obtained 677 articles. In conducting this review, this initial set formed the base to be reviewed in order to shortlist those for which our parameters were met. In the first phase, all articles underwent an initial screening based on their titles to ensure consistency and relevance to our research goals. Following this initial screening and subsequent removal of duplicates, 136 were selected for further evaluation.

**Figure 1:**
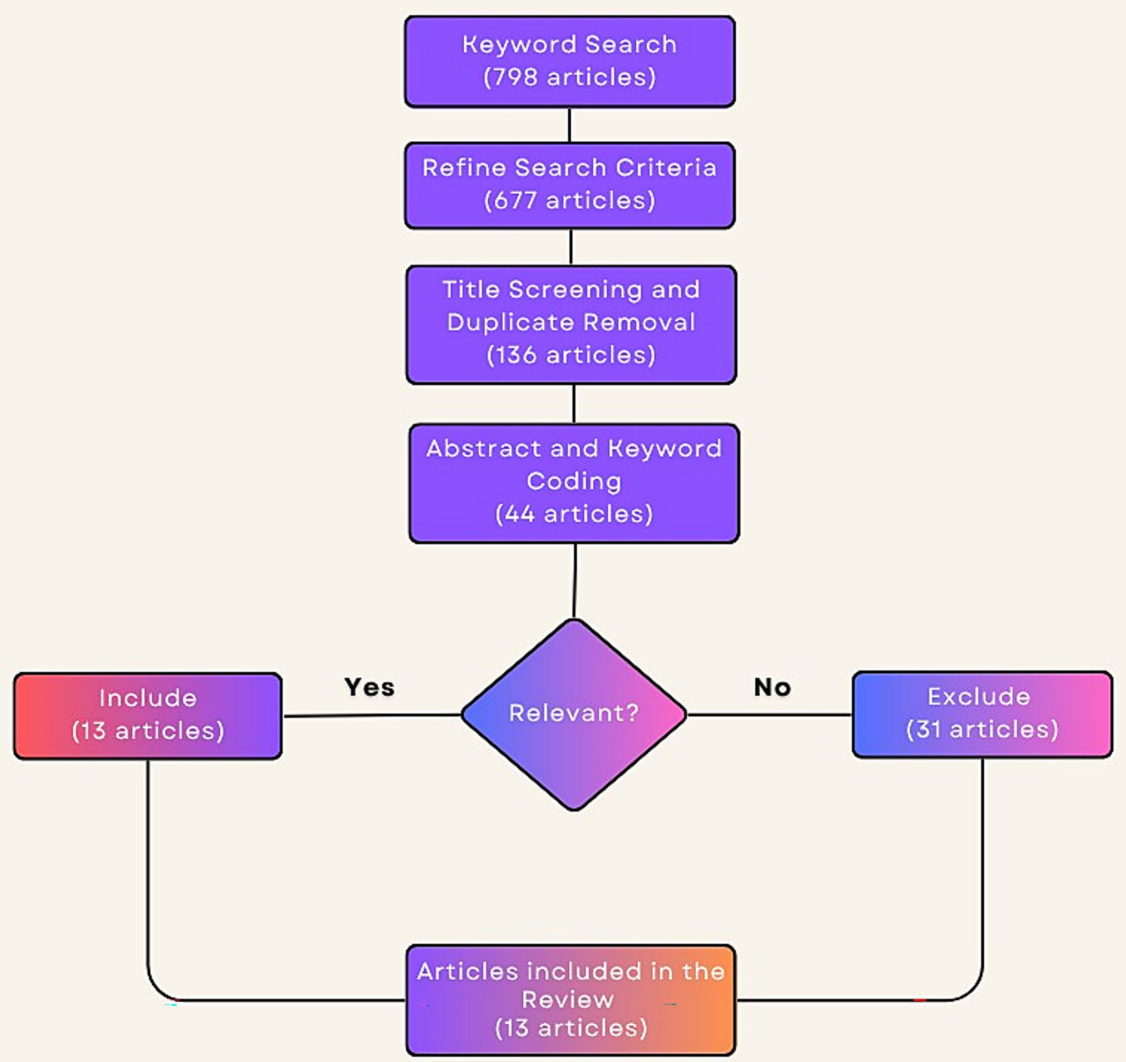
Flowchart for the retrieval and screening of the articles.

Initial coding was done in the second stage by preliminary analysis of the abstracts, keywords, and if judged necessary, the introductory sections of the articles. This yielded a sub-set of 38 articles closely matching our current focus. Finally, in the final screening stage, all remaining articles were to be fully screened to establish how relevant and applicable they actually were to our scope of study. Consequently, a final set of 13 articles that best contributed to our research objectives and met the inclusion criteria were identified and retained (Figure 1). To synthesize findings from the included studies, four extractors independently reviewed the full texts and extracted relevant data. We then conducted a narrative synthesis, conceptually grouping findings into recurring domains such as sensor modalities, implementation settings, target behaviors (e.g., agitation and aggression), and personalized detection models. The themes were developed through an inductive and iterative process, allowing for a structured yet flexible integration of findings across studies with varying designs and methodological approaches.

## 3 Findings

### 3.1 Understanding agitation

Understanding the root causes of agitation is crucial for recognizing, comprehending, and ultimately addressing its manifestations. An individual’s behavior is shaped by intricate interactions between their internal and external environment. When a person can successfully convey their internal patterns to the external world, it provides an opportunity to express their thoughts, feelings, concerns, and needs. This communication allows the recipient to respond appropriately, according to their individual interaction of internal and external processes. However, where there is an impairment to the communication, as in the conditions of dementia and autism, the ability to articulate internal experiences is impaired. An inability to express internal needs leads to a state of distress, which provides a fertile ground for agitation and other problems to set in.

Agitation is characterized by inappropriate verbal, vocal, or motor activities that external observers may not attribute to a specific need. The Unmet Needs Model (12) aligns with these observations, proposing that as dementia progresses, the effective communication of needs diminishes, concurrently impacting the ability for self-provision (13). Stokes (14) similarly describes agitation as an active attempt by the individual to express an unmet need, whether physiological or psychological.

According to Cohen-Mansfield et al. (15), needs may include physical discomfort, mental unease, the desire for social interaction, discomfort in the environment, or insufficient stimulation. Caregivers and surroundings may inadequately address these needs, resulting in agitated behaviors like pacing, aimed at relieving boredom, or repetitive vocalizations serving as a form of communication. Interestingly, the researcher uncovered that specific unmet needs correspond to distinct agitated behaviors. Social needs, pain, and discomfort, for instance, align with verbal agitation, whereas boredom and the need for stimulation are associated with physically non-aggressive agitated behaviors. Given the significant distinctions mentioned above, this could mark the point where technology-assisted early detection of agitation brings precision to the forefront, informing caregivers about the nature of distress and providing potential insights into the specific unmet needs of the individual.

#### 3.1.1 Subtypes of agitation

The various subtypes of agitation in individuals with neurological impairments, especially those who do not communicate effectively, can provide an extremely important perspective for the caregivers and healthcare professionals. Recognizing that agitation is not a uniform experience but rather a spectrum of behaviors, facilitates a better evaluation of the individual’s needs and triggers. With respect to agitation in elderly individuals, Cohen-Mansfield’s categorization provides a comprehensive framework, classifying these behaviors into four distinct subtypes (16):

Verbally non-aggressive type: Persistently seeking attention or assistance, expressing discontent, conveying negativity, and repeating sentences or queries.

Verbally aggressive type: Engaging in loud screaming, uttering profanities, showing verbal aggression and making unusual sounds.

Physically non-aggressive type: Engaging in repetitive movements, consuming unconventional substances, mishandling objects, attempting to relocate, wandering aimlessly, purposefully falling and displaying restlessness.

Physically aggressive type: Causing harm to oneself or others, throwing objects, scratching, grasping or grabbing, forceful pushing, spitting, kicking, biting and engaging in inappropriate sexual behavior.

#### 3.1.2 Neurobiology of agitation

Examining the neurobiology and neurochemistry of agitation has the potential to enrich our comprehension of this phenomenon. More specifically, in the case of Alzheimer’s disease (AD), agitation may stem from deficiencies in the serotonergic system, particularly involving receptor subtypes such as 5-HT1A and 5-HT1B. Notably, dopamine levels in agitated AD patients appear to be relatively sustained. The noradrenergic system, associated with arousal, may contribute through compensatory overactivity. Despite indications of participation, the functions of the cholinergic and GABAergic systems in agitation necessitate further exploration. You may insert up to 5 heading levels into your manuscript as can be seen in “Styles” tab of this template. These formatting styles are meant as a guide, as long as the heading levels are clear, Frontiers style will be applied during typesetting.

**Figure 2:**
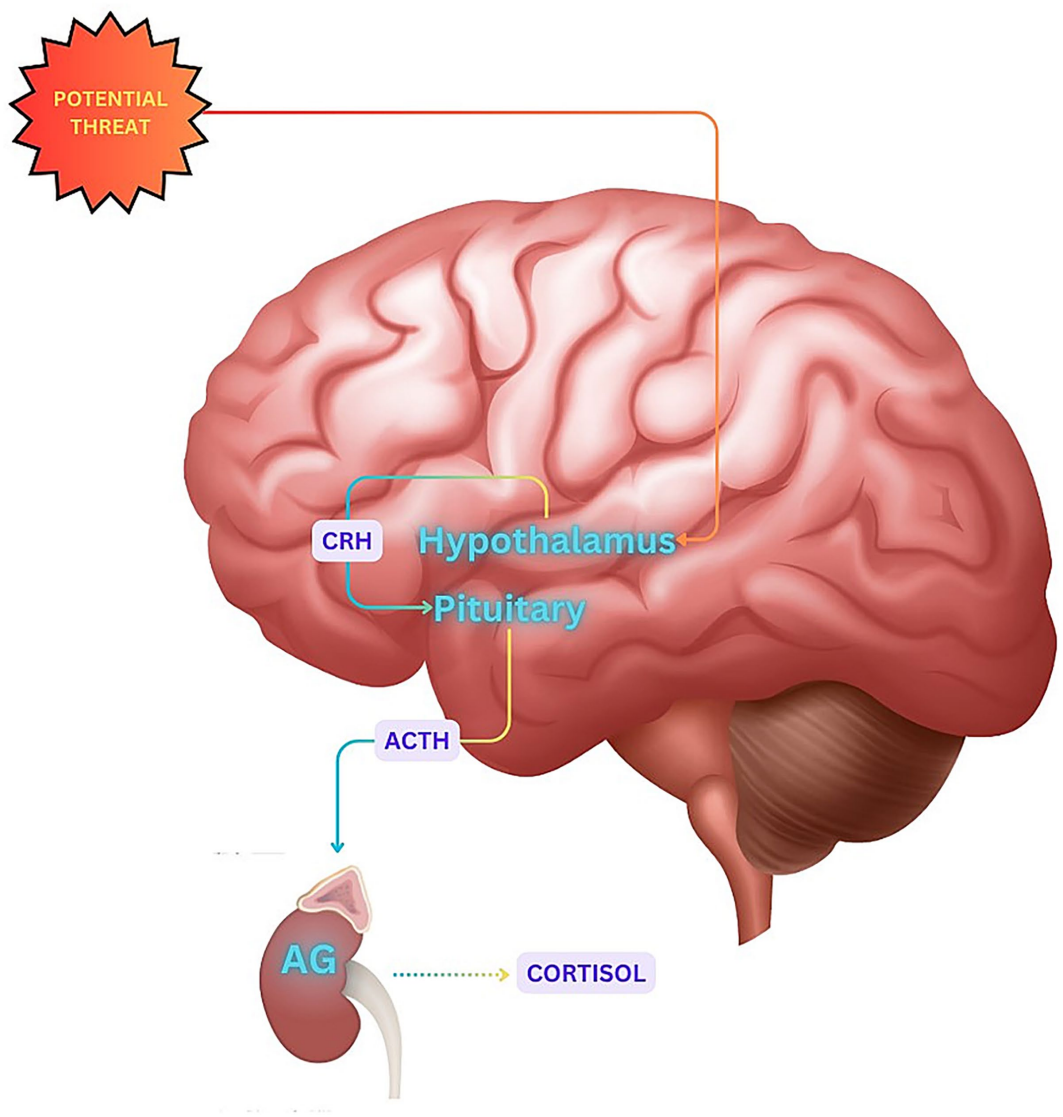
Perceived threat misinterpretation and HPA axis activation. CRH, Corticotropin-releasing hormone; ACTH, adrenocorticotropic hormone; AG, adrenal gland.

Liu et al. (17) provide a neuropsychological perspective on how patients with AD often interpret potential threats in a way that amplifies their emotional arousal into states of agitation. Such exaggerated valuation of the environment can activate the HPA axis and lead to an oversecretion of cortisol (Figure 2). This stress reaction might further be associated with the release of catecholamines and changes in neurotransmitters associated with agitation, such as glutamate and acetylcholine.

**Figure 3:**
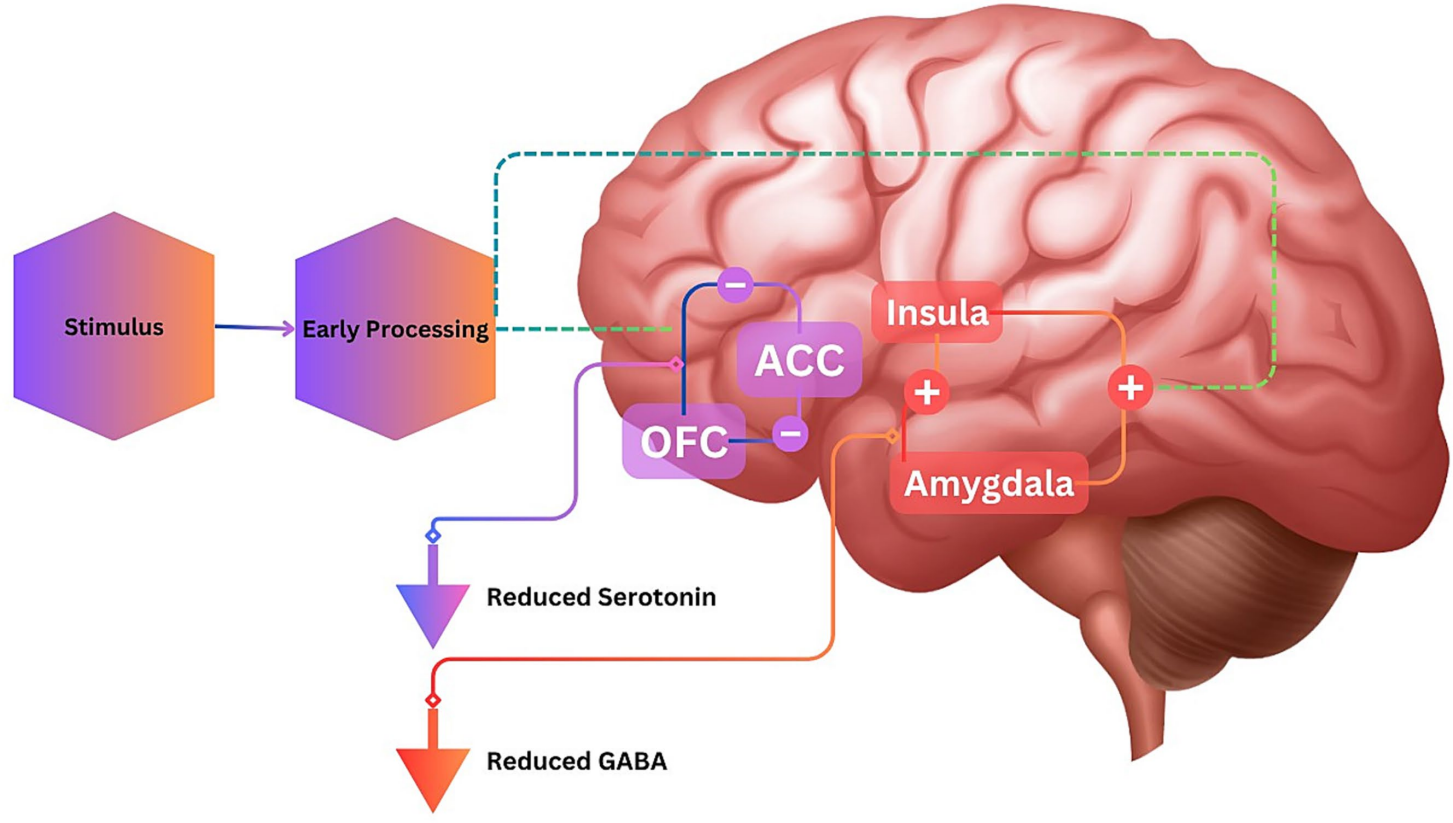
Neuromodulators regulating aggression. The diagram illustrates how reduced levels of serotonin lead to insufficient processing in the orbitofrontal cortex (OFC) and anterior cingulate cortex (ACC), resulting in impaired top-down control of behavior. Additionally, reduced GABA levels cause hyperactivity in the amygdala, contributing to emotional hypersensitivity and heightened aggressive responses.

As shown in Figure 3, aggressive states are typically associated with diminished levels of serotonin and GABA, along with other crucial neuromodulators necessary for the top-down control of limbic activation. Research indicates that specific abnormalities in brain structural circuitry and the regulation of these neuromodulators may collectively contribute to an increased susceptibility to aggression. Consequently, agitation in dementia patients could hypothetically arise from compromised top-down cortical control over impulsive behaviors or irregular bottom-up impulses originating from limbic regions. Interestingly, the pathways and circuits involved in fear activation significantly overlap with those implicated in aggressive responses (18, 19).

**Figure 4:**
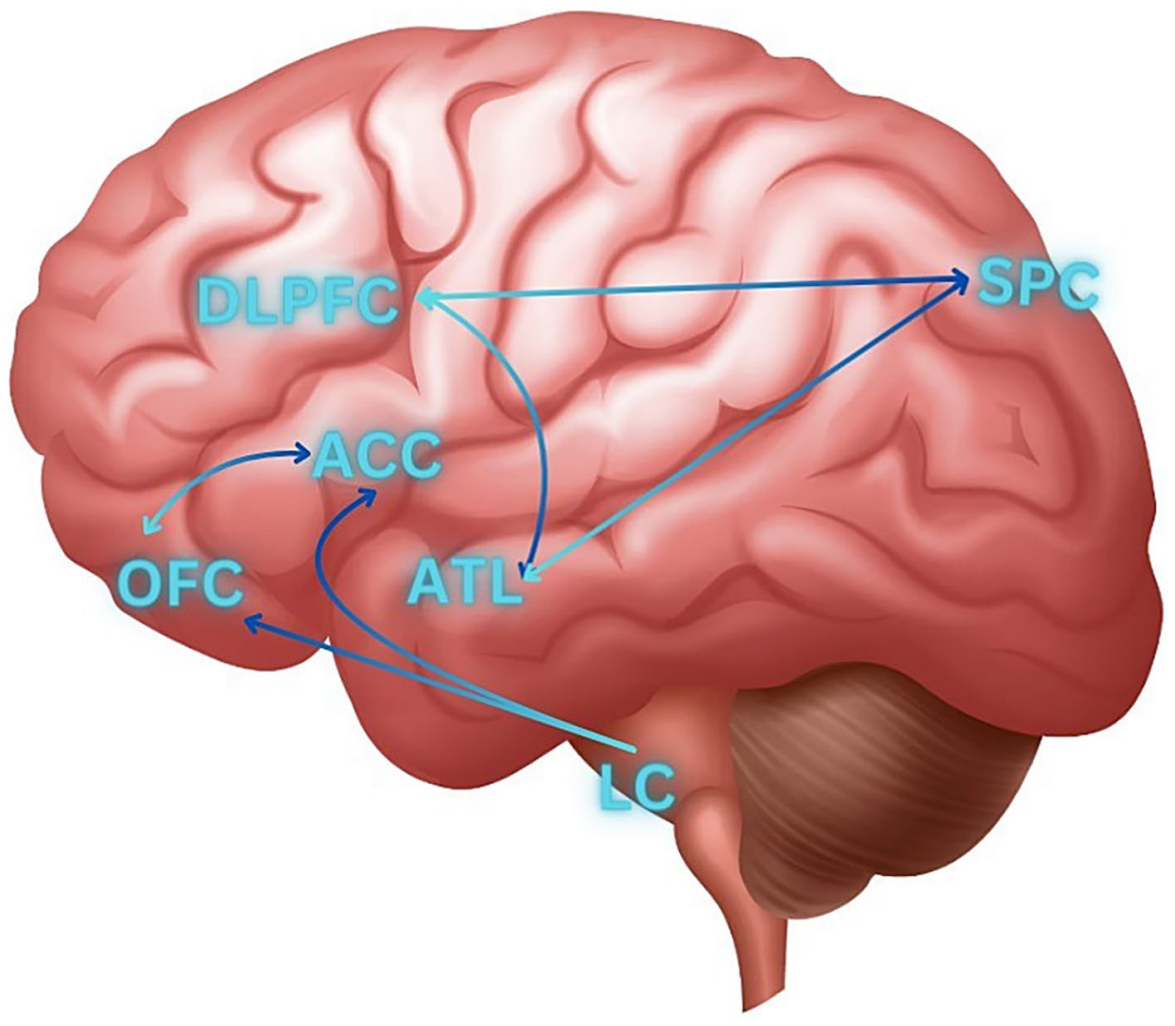
Brain regions and pathways involved in agitation. ACC, Anterior cingulate cortex; ATL, anterior temporal lobe; DLPFC, dorsolateral pre-frontal cortex; LC, locus coeruleus; OFC, orbitofrontal cortex; SPC, superior parietal cortex.

In this context, the amygdala plays a crucial role, housing adrenoreceptors and dopamine D2 receptors. Furthermore, direct projections onto amygdalae nuclei from brainstem areas, specifically the ventral tegmental area and locus ceruleus, intensify amygdala excitation, consequently increasing emotional reactivity (20). This mismatch between stimuli and the elicited emotional reaction, is additionally supported by Carrarini et al. (21), through a different system associated with frontal lobe dysfunctions, specifically characterized by abnormal activation of the orbitofrontal cortex (OFC) and anterior cingulate cortex (ACC). Diminished blood flow in the dorsolateral prefrontal cortex (dlPFC), superior parietal cortex (SPC), and anterior temporal lobe (ATL) is postulated to play a role in generating abnormal emotional responses in dementia. According to the researchers, a dysfunctional interaction among these cerebral regions may provide clarification for abnormal behavioral responses (Figure 4).

Adding to all the aforementioned complexities, the typical communication impairments in individuals with AD can further convolute the manifestations of distress.

### 3.2 Measurement of agitation

#### 3.2.1 Neuropsychiatric assessments

In the fields of psychiatry and clinical neuropsychology, the examination of challenging behaviors often involves the utilization of assessment scales to assess the extent or nature of agitation based on common manifestations of such behaviors, as described earlier (see Table 2). These scales can be very helpful clinically in many ways. However, research findings and practical clinical experiences indicate that using these tools can, in some cases, be time-consuming. In addition, the complexity of certain states of agitation adds challenges to precise measurement. This phenomenon has the potential to further contribute to the susceptibility of observer bias in assessments, as suggested by previous studies (22).

**Table 2:** Neuropsychological and psychiatric assessments of agitation in psychiatric settings.

Assessment tool	Creator(s) and year	Description
Agitation Severity Scale (ASS)	Strout (2014) (37)	The ASS measures the severity of agitation in psychiatric patients, providing a quantitative assessment to guide treatment decisions.
Behavioral Activity Rating Scale (BARS)	Swift et al. (2002) (38)	BARS evaluates the level of agitation based on observable behaviors, aiding in the assessment and management of agitation in various clinical settings.
Brief Agitation Measure (BAM)	Ribeiro et al. (2011) (39)	BAM is a brief tool designed to quickly assess agitation severity in psychiatric patients, facilitating rapid evaluation and intervention.
Clinical Global Impression Scale for Aggression (CGI-A)	Huber et al. (2008) (40)	CGI-A provides a clinician’s overall impression of the severity of aggression in psychiatric patients, assisting in treatment planning and monitoring.
Cohen-Mansfield Agitation Inventory (CMAI)	Cohen-Mansfield et al. (1986) (41)	CMAI assesses various forms of agitation, aiding in the evaluation and management of agitation in dementia and psychiatric patients.
Neuropsychiatric Inventory (NPI)	Cummings et al. (1994) (42)	NPI assesses a wide range of neuropsychiatric symptoms, including agitation, in dementia patients, facilitating comprehensive evaluation and treatment planning.
Overt Aggression Scale (OAS)	Yudofsky (1986) (43)	OAS measures the severity of overt aggression, including agitation-related behaviors, assisting in the assessment and management of aggressive behaviors.
Overt Agitation Severity Scale (OASS)	Yudofsky et al. (1997) (44)	OASS provides a structured assessment of agitation severity in psychiatric patients, aiding in the monitoring of treatment response and symptom progression.
Pittsburgh Agitation Scale (PAS)	Rosen et al. (1994) (45)	PAS assesses the severity of agitation in psychiatric patients, providing a standardized measure for evaluating agitation-related symptoms.
Positive and Negative Syndrome Scale Excited Component (PANSS-EC)	Kay et al. (1987) (46)	PANSS-EC evaluates the excited component of psychosis, including agitation, assisting in the assessment and monitoring of psychotic symptoms.
Rating Scale for Aggressive Behavior in the Elderly (RAGE)	Patel and Hope (1992) (47)	RAGE assesses aggressive behaviors in elderly patients, including those with dementia, aiding in the evaluation and management of aggressive tendencies.
Richmond Agitation-Sedation Scale (RASS)	Sessler et al. (2002) (48)	RASS measures agitation and sedation levels in critically ill patients, providing a standardized assessment tool for evaluating sedation and agitation states.
Staff Observation Aggression Scale (SOAS)	Palmstierna and Wistedt (1987) (49)	SOAS is used by staff to observe and document aggressive behaviors in psychiatric patients, facilitating the management and prevention of aggression incidents.

Considering the limitations of these traditional approaches, this study seeks to explore contemporary approaches using technological advancements toward early agitated behavior detection. The next section describes the main sensing paradigms on which our discussion is based, setting the stage for an in-depth review of studies that use these modalities.

#### 3.2.2 Wearable sensors

Wearable sensors have emerged as important tools in the domain of dementia care, by enabling real-time detection, diagnosis, and monitoring of various behavioral and physiological aspects related to the condition. Such devices, designed to be worn on the body or clothing, provide the facility of non-invasive monitoring of physiological data, that could help in the timely detection of symptoms and their intervention strategies. In the context of the disorders examined in this paper, a number of prior studies have, for instance, assessed the validity of actigraphs in quantifying motor agitation-related limb movements in PwD (23). However, it’s crucial to acknowledge that expressions of agitation extend beyond mere motor actions and may encompass emotions such as anger, distress, or vocal outbursts. These behaviors can elicit changes in physiological markers like skin conductivity and heart rate variability. These metrics may serve as direct indicators of autonomic nervous system arousal, potentially acting as digital biomarkers that can reveal an individual’s internal state of stress or pain before it becomes visible to the naked eye. Typically, precursor stages precede any escalation into agitated behavior due to unmet needs. This is where common physiological sensors like blood volume pulse, skin conductance, and skin temperature are commonly employed to identify or even forecast agitation in PwD during these early stages. Ultimately, these physiological indicators may allow clinicians and researchers to detect subtle shifts from a person’s usual baseline and to recognize an emerging state of distress rather than waiting for a full behavioral crisis to manifest. In this way, wearable sensors have the potential to act as an objective early warning system that can support timely interventions.

#### 3.2.3 Environmental and contextual sensors

In addition to direct wearable measurements, environmental sensors may provide contextual information that can influence or help interpret agitation in PwD. Like the general population, the living environment for PwD can have a profound effect on mood and behavior and may even escalate states of agitation. Technologies such as accelerometers and ambient devices, including passive infrared and motion sensors, may provide helpful insights into environmental factors like noise levels, lighting, and temperature fluctuations. This is particularly critical given that agitation may often stem from identifiable external stressors, such as an overly warm room or excessive noise, which can be objectively quantified by these sensors. For example, novel sensors such as smart power plugs and door sensors not only can provide important contextual data but also complement the collection of physiological information, helping caregivers gain a more comprehensive understanding of agitation triggers and thus develop tailored interventions for each individual (24). Furthermore, the adoption of multiple sensors in caregiving practices might help in developing a more detailed and personalized comprehension of the complicated needs of PwD. By linking environmental metrics directly to behavioral episodes, these technologies can provide a clearer understanding of the contextual triggers of agitation. Continuous tracking of spatial patterns, including wandering or disrupted night-time routines via motion sensors, could allow caregivers to distinguish between agitation arising from intrinsic factors and that provoked by external stressors. The knowledge gained through such technological tools could possibly enable caregivers to develop a more supportive and friendly atmosphere that could quite dramatically improve the well-being and quality of life for PwD.

#### 3.2.4 Artificial intelligence

Recently, AI has emerged as a promising intervention model for early detection and management in healthcare. More specifically, AI algorithms can be used to analyze data gathered from wearable devices, environmental sensors, or other monitoring systems. An interesting example of the use of AI in dementia care is referred to as digital phenotyping. This involves the use of AI algorithms in the analysis of sensor data for the description of unique patterns of behavior in a quantitative manner. This strategy can help in the timely identification and categorization of the symptoms of dementia, hence allowing interventions and care plans that are tailor-made for each individual. Central to this process is the ability of ML models to establish personalized baselines for each individual, rather than relying on generic population thresholds. By learning an individual’s typical autonomic and behavioral patterns, these systems can detect subtle deviations that may indicate emerging agitation. This strategy can help in the timely identification and categorization of the symptoms of dementia, hence allowing interventions and care plans that are tailor-made for each individual (25). Advanced temporal modeling approaches, including recurrent neural networks, can extend this capability by analyzing sequential patterns over time. Such models may forecast potential agitation episodes minutes—sometimes tens of minutes—in advance, thereby creating a predictive window that is critical for early, non-pharmacological intervention. Therefore, the application of artificial intelligence technology in this context enables healthcare practitioners to augment real-time monitoring and increase the precision of symptom identification, ultimately fostering improved outcomes for individuals diagnosed with dementia.

#### 3.2.5 Multimodal approach

Within the field of dementia care itself, there has been a growing emphasis on multimodal approaches to identifying agitation, which aim to integrate diverse sensor data streams to offer more holistic and nuanced insights into agitated behaviors. The strategy involves the combination of various sensor types for monitoring a broad range of physiological reactions and behavioral activity in PwD. This fusion of data streams allows for cross-validation of signals, significantly reducing false positives; for example, high heart rate combined with vigorous motion might indicate exercise, whereas high heart rate with low motion and high skin conductance is more suggestive of psychological distress. Indeed, studies suggest that the integration of physiological sensor data with accelerometer information and input from different ambient sensors showed a significant improvement in the recognition of different patterns of agitation in PwD. The results and findings illustrate the benefits of using multimodal methodologies compared to single-sensor approaches, aligning with the existing literature supporting the use of multiple sensor modalities in increasing agitation detection accuracy (26, 27). Such observations may justify the potential of multimodal sensing approaches for a comprehensive agitation assessment to provide tailor-made care and interventions for PwD in both clinical and residential settings, capturing both the internal physiological state and the external context to maximize the reliability of early warning systems.

#### 3.2.6 Studies applying sensor technology for early identification of agitation

Based on the integrated insights from the previously discussed sensor technologies, our literature review identified several research studies (see Supplementary File S2) that have used wearable technology and multimodal systems in addressing the complicated issues related to monitoring and managing behavioral symptoms in PwD. In this chapter, we present these studies in chronological order, highlighting the progression of sensor-based approaches over time and the insights each has contributed to understanding agitation detection and management.

In 2017, Salekin et al. (28) presented the Detecting Agitated Vocal Events system (DAVE), which used acoustic signal processing along with textual inference to detect verbal agitations of older adults with dementia. Their approach achieved high detection accuracies for several verbal behaviors, including asking for help and verbal sexual advances, with high accuracies of 93.45 and 91.69%, respectively Moreover, it was an effective system in agitation recognition among mumbling dementia patients, and it is invaluable assistance for healthcare professionals in the identification and treatment of such behaviors.

Building on this, Nesbitt et al. (29) conducted a study using wearable sensors to track the levels of agitation among dementia patients living in memory unit assisted living facilities. This study had a sample size of eight residents who were diagnosed with Behavioral and Psychological Symptoms of Dementia (BPSD). All patients used wearables that included tri-axial accelerometers to measure limb movement, heart rate monitors, microphones for audio data, and bluetooth devices for location within the facility.

All these sensors, combined, measured different parameters such as limb movement, heart rate, audio, and location within the facility. The results of the trial showed it was indeed possible to use wearable sensors for the early detection of agitation in dementia patients. They seemed to provide very important information on agitation patterns, enabling timely intervention and support from carers. Most importantly, the researchers did show a correlation between the human observations and the data obtained from the sensors, thus affirming the effectiveness of wearable technology in detecting and monitoring agitation among PwD.

In a deeper look into what sensor technology could bring forth, Bankole et al. (30) elaborated on a pilot study concerning dementia care that involved 12 dyads, each composed of a person with dementia (PWD) and their caregiver. The focus of the study was the use of the Behavioral and Environmental Sensing and Intervention for Dementia Caregiver Empowerment system (BESI), integrating body-worn inertial sensors, including TEMPO, Shimmer, and Pebble, along with environmental sensors installed in the house. Together, the components support continuous, non-invasive assessments of agitation while constantly monitoring the environmental context in which the behavior occurs in order to detect early cues of agitation and possible triggers of agitation. The researchers have stated that this integrated approach allowed for real-time monitoring of agitation events in PwD and enabled early detection as well as interventions while reducing the burden of caregivers. Notably, this study confirmed the relationship between dementia-related agitation and environmental factors such as temperature and humidity levels, thus establishing important insights into agitation triggers. The authors point out that the preference of home-based monitoring systems such as BESI among PwD and caregivers really emphasizes the need for non-invasive and continuous assessment and monitoring that will finally blend with daily life.

In the same year, Yeung et al. (31) conducted a pilot study with residents with dementia in memory care facilities who had a score above 50 on the Cohen-Mansfield Agitation Inventory. Employing a heterogeneous ensemble of sensors, including wearable actigraphy devices (Actiwatch Spectrum), bed pressure mats (Emfit), environmental sensors (Thunderboard Sense 2–SLTB004A), and ambient sensors (NYCE Sensors), the study set out to monitor participants’ activity levels, observe sleep metrics, and analyze environmental influences. Synthesizing such sensor data enabled the identification of agitation by analyzing participant movement data, sleep metrics, and environmental parameters. This study demonstrates that, using multimodal sensing, agitation in dementia can indeed be monitored and thus predicted in care settings. This study further reported associations of environmental factors with nighttime agitation, such as humidity, and increased activity levels before agitation, which underline potential correlations worthy of further investigation.

In a very similar line of thought, Spasojevic et al. (27) conducted longitudinal research, involving 17 participants from the Specialized Dementia Unit at Toronto Rehabilitation Institute over a 600-day period. They used a multimodal sensor system to extensively monitor agitation and aggression in PwD, covering both physical movement and behavioral cues for effective detection and intervention. The study highlighted the significance of personalized ML models, which outperformed generic models, thus emphasizing the importance of tailoring detection algorithms to individual characteristics. Combining physiological and visual cues through multi-modal features-based models has demonstrated superiority over single-sensor models in the detection of agitation in PwD. These findings have been able to indicate the importance of personalized classification models in addressing the unique agitation patterns of individual participants, hence serving as important insights for future research and clinical practice.

In the same year, Hekmati-Athar et al. (32) used data from the BESI study to perform a forecasting study that distinguished between periods of agitation and non-agitation in the home of a dementia dyad. They recorded various environmental data, ambient acoustic noise level, illuminance, temperature, atmospheric pressure, and humidity level, to train a deep learning (DL) model called long short-term memory (LSTM) for the prediction of agitation episodes in persons with dementia. The model, which was designed to receive the input of sensory data from the past 30 min, achieved noteworthy results, with 98.6% accuracy and 84.8% recall in predicting agitation episodes among Alzheimer’s disease dyads residing in the community. These results prove that the potential of a data-driven DL approach—particularly LSTM—is promising for predicting agitation events through environmental cues, opening new avenues toward early intervention and de-escalation strategies for dementia care settings.

During the same period, a study team led by Iaboni et al. (25), conducted an initial longitudinal study of 20 older adults aged 55 and above admitted for dementia-related behavioral and psychological symptoms (BPSD) to the Specialized Dementia Unit (SDU), Toronto Rehabilitation Institute (TRI). Various sensors were used in the study, such as accelerometers, blood volume pulse sensors, electrodermal activity sensors, and skin temperature sensors to collect data on Behavioral and Psychological Symptoms of Dementia (BPSD). The researchers used the collected data to create algorithms for real-time detection of agitated behaviors, using methods known as digital phenotyping, which quantifies individual human phenotypes *in situ* using data from personal digital devices. The findings showed that ML models improved significantly on the classification of symptoms, obtaining a median area under the curve of 0.87. They emphasized that combining sensor data with machine learning algorithms brought about the capability of minute-by- minute symptom detection, offering a possible step toward more advanced real-time monitoring and more timely, tailored interventions.

A research team led by Davidoff et al. (26), extended the scope of sensor-based interventions by conducting a pilot study in 2022 on a multimodal sensor system measuring the psychological, contextual, and physiological parameters of dementia patients. The study included PwD classified into Tier 6 and Tier 7 of the BPSD care model and were admitted to a specialized neuropsychiatric ward. A general multimodal sensor system was used for this study, which had several types of sensors: an environmental sensor known as Chill+, which measures the temperature and humidity; a LYS button sensor to measure the light level; a tracker to measure the BLE localization; an under-mattress sensor using EMFIT QS-based monitors of physiological measures; a Meta-tracker to capture environmental parameters; and a Nokia phone for audio data collection. By synchronizing data from the sensors above, the study attempted to develop a detailed profile of each patient’s living environment and agitation state. Various factors were considered in this methodology for identifying agitation triggers, such as contextual elements, physiological conditions, and psychological indicators. This multimodal sensing approach in the clinical setting demonstrated the feasibility of performing a highly detailed quantification and characterization of patients’ living conditions. Real-time monitoring of such parameters is thus capable of uncovering a variety of possible agitation triggers and allows a better understanding of the daily pattern variation of agitation.

In their recent observational study, Khan et al. (24) used computer vision to approach the detection of agitation in PwD. Using visual information captured by video recording cameras, the authors created a sample of 20 participants from the Specialized Dementia Unit at TRI in Toronto, Canada, with a mean age of 80.5 years. The dataset was used for the training of a handcrafted spatio-temporal convolution autoencoder (3DCAE), adapted to distinguish agitation events from normal activities. After a dense training on a dataset comprising 24 h of normal behaviors, the algorithm of 3DCAE was tested further on an 11-h-long dataset that included both agitation events and normal activities. The researchers suggest that the algorithm showed a great capacity for accurately identifying agitation, effectively spotting anomalous behavioral patterns within the video recordings. Another very important methodological commitment within the research has been related to privacy. In order to protect the identity of the individuals appearing in the video data, measures to hide and protect their identities were carried out to meet the ethical requirements of health data governance. The authors argue that their project delivers an integrated solution for real-time agitation monitoring and detection, with a minimum of intervention by staff and residents; the computer vision algorithm really appears to be a very promising method in this respect for improving care delivery and intervention strategies in dementia care facilities.

Another technological approach to agitation monitoring in dementia care has been proposed in the study by Bafaloukou et al. (33), which utilized ML techniques for in-home monitoring of agitation episodes in PwD. The researchers developed an interpretable framework based on passive sensor data, enabling remote and continuous assessment of behavioral changes associated with agitation. The study collected longitudinal data from 63 participants over 32,896 person-days, using in-home monitoring devices that captured activity levels, sleep disturbances, environmental parameters such as light and temperature, and physiological signals like respiratory rate. The study implemented a Light Gradient-Boosting Machine (LightGBM) model, which demonstrated a sensitivity of 71.32% and a specificity of 75.28% in identifying agitation episodes. A traffic-light stratification system further improved specificity to 90.3%. The implemented framework appeared to improve model interpretability, revealing key agitation predictors such as low nocturnal respiratory rate, heightened alertness during sleep, and increased indoor illuminance. Additionally, the researchers introduced an interactive decision-support tool, allowing clinicians to simulate non-pharmacological interventions in silico before applying them in real-world settings. For example, modifying indoor lighting and adjusting temperature settings emerged as feasible strategies to mitigate agitation risk, aligning with recommendations for personalized dementia care.

Finally, another exploration of wearable technology comes from Milbotix SmartSocks, which integrate sensors to monitor physiological data from the foot and ankle, offering a potentially more comfortable alternative to traditional wearable devices for PwD (34). The researchers noted that this foot-worn wearable device was rated lower in terms of comfort issues compared to wrist- and chest-worn devices. The accuracy of the pulse rate estimates, as derived from photoplethysmography (PPG) and validated by a Shimmer 1-lead electrocardiogram (ECG), had an average absolute error of less than 5 beats per minute (bpm). The PPG signal quality classification by neural networks achieved an accuracy of around 95%. In a preliminary feasibility study, PwD exhibited reduced agitation levels, according to the Abbey Pain Scale (APS) and the Neuropsychiatric Inventory (NPI), following 2 weeks of wearing SmartSocks. The results imply that SmartSocks may prove to be an effective early warning of distress with a comfortable and accurate option for conventional wearable devices, and at the same time, provide valuable information for personalized care interventions.

**Figure 5:**
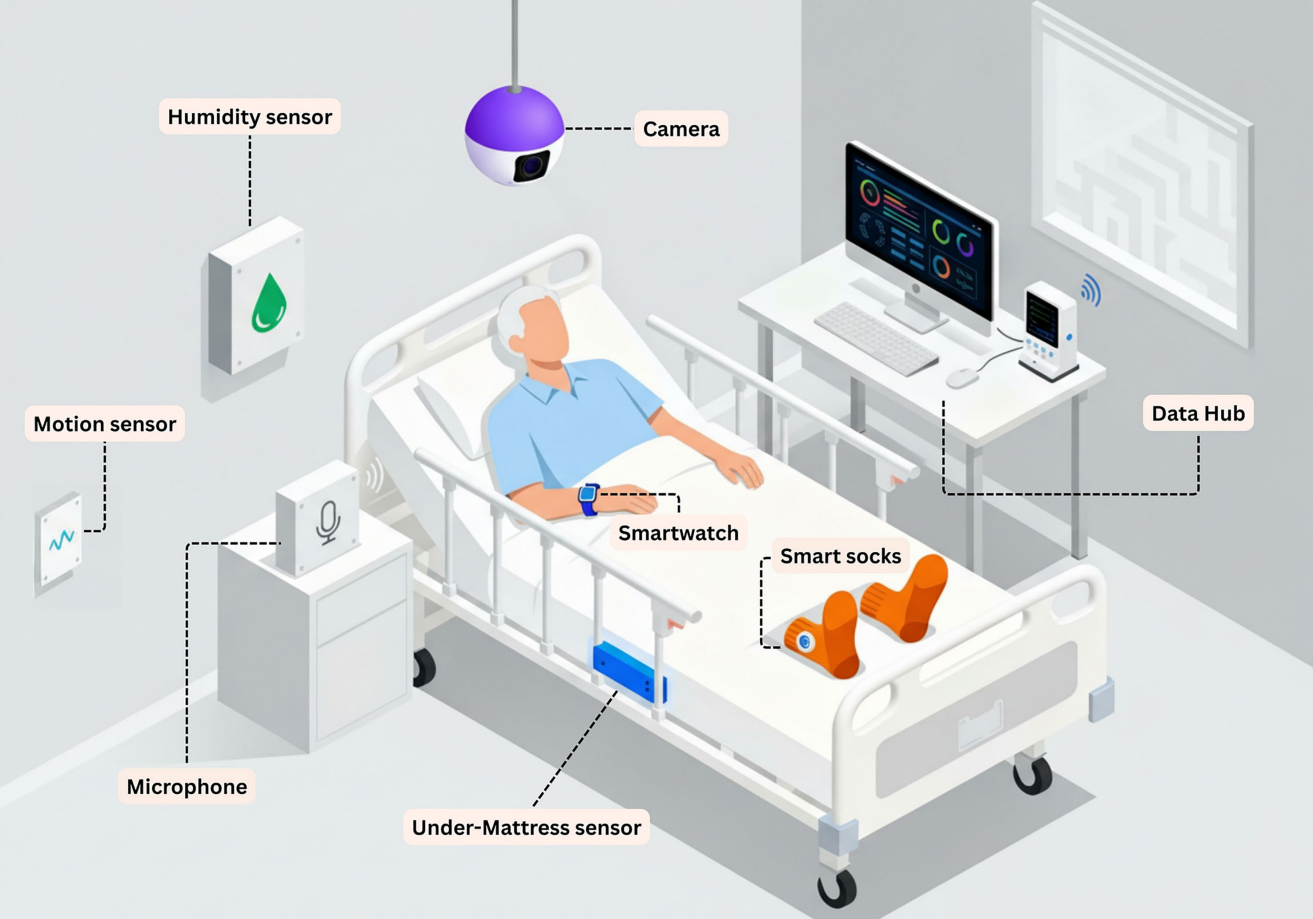
Prototype schematic of a multimodal sensor configuration within a care facility room. The figure illustrates a conceptual configuration synthesizing approaches observed across the included studies. Wearable sensors monitor physiological parameters and activity. Environmental sensors capture contextual data on humidity, temperature, and motion. The under-mattress sensor monitors sleep–wake patterns and bed based activity. Computer vision enables behavioral observation. All data streams integrate to a central data hub for real-time analysis and caregiver alerting.

As a closing note, to complement these study descriptions, we present a hypothetical schematic of a multimodal sensor configuration within a care facility room (Figure 5). While not all of these sensors would necessarily be deployed simultaneously in a real-world setting, they are presented together here to offer a comprehensive overview of potential configurations.

### 3.3 Thematic synthesis across the included studies

While the preceding section outlined the chronological progression of sensor-based agitation detection research, a synthesis of findings across the included studies reveals several cross-cutting themes. Beyond individual study outcomes, critical domains emerge that characterize the current state of the field, ranging from technical performance to practical implementation in real-world care settings.

#### 3.3.1 Variations in detection performance across sensor systems

Performance metrics appeared to vary across the reviewed studies, likely reflecting differences in sensor modalities and algorithmic approaches. Single-modality systems, such as the acoustic-only approach by Salekin et al. (28), seemed effective for specific verbal behaviors (reporting 93.45% accuracy for “asking for help”) but appeared limited to vocal manifestations. Across the included studies summarized in Supplementary File S2, multimodal systems, integrating two or more distinct sensor streams, were more common than single-modality systems; studies using multiple features derived from a single sensing domain (e.g., environmental variables only) were classified as single-modality approaches. Eight studies employed multimodal sensing approaches, whereas three relied primarily on a single sensing modality. Reported performance was heterogeneous and therefore does not support aggregation into a single overall accuracy estimate. Nevertheless, among the single-modality studies reporting accuracy, task-specific performance ranged from 91.69 to 98.6% (including 93.45 and 91.69% for detection of specific vocal events and 98.6% accuracy with 84.8% recall for short-horizon forecasting, as reported by Hekmati-Athar et al. (32) using environmental data from the BESI study). In contrast, multimodal studies more often evaluated broader detection problems using AUC and/or sensitivity and specificity; for example, Spasojevic et al. (27) and Yeung et al. (31) reported improved performance when physiological measures (e.g., blood volume pulse and electrodermal activity) were combined with contextual information, Iaboni et al. (25) reported a median AUC of 0.87, and Bafaloukou et al. (33) reported 71.32% sensitivity and 75.28% specificity using in-home sensors, with a traffic-light decision-support stratification improving specificity to 90.3%. Multimodal systems were typically designed to combine complementary physiological and contextual signals to support performance stability across individuals and care contexts, rather than optimizing accuracy for narrowly constrained outcomes.

#### 3.3.2 Influences of reference measures and contextual Bias

A pervasive challenge identified across studies is the reliance on subjective “ground truth” for training algorithms. Most studies validated their sensor data against human observation. As noted in Nesbitt et al. (29), while correlations exist between human observation and sensor data, this reliance introduces susceptibility to observer subjectivity. Furthermore, environmental confounders often complicate this picture; studies like Bankole et al. (30) and Yeung et al. (31) demonstrated that environmental factors (temperature, humidity) correlate with agitation, suggesting that algorithms must distinguish between agitation driven by internal distress versus reactions to uncomfortable environmental conditions.

#### 3.3.3 Technical challenges in physiological sensing

The transition to real-world deployment introduces significant challenges regarding physiological data quality. Signals such as electrodermal activity and heart rate variability are sensitive to artifacts. Steer et al. (34) addressed data quality in wearable form factors by validating pulse rate estimates from “SmartSocks” against ECG, achieving less than 5 bpm error and 95% accuracy in signal quality classification. The need for robust artifact rejection is implicit in multimodal systems like those of Spasojevic et al. (27), which combine accelerometry with physiological signals to likely help distinguish motion-induced artifacts from genuine autonomic arousal.

#### 3.3.4 Implementation feasibility and user acceptance

Adherence to wearable technology remains a critical barrier. Steer et al. (34) explicitly highlighted that SmartSocks had lower comfort concerns compared to wrist- and chest-worn devices, addressing the challenge of device tolerance in dementia populations. Successful long-term deployments, such as the 600-day monitoring period achieved by Spasojevic et al. (27), suggest that consistent monitoring is feasible. Other studies, like Yeung et al. (31) and Davidoff et al. (26), utilized passive sensing (under-mattress sensors, ambient sensors) to maintain monitoring without requiring active patient compliance, thereby overcoming some adherence barriers associated with wearables.

#### 3.3.5 Applicability across care environments

The implementation context significantly influences both system design and generalizability. Studies conducted in institutional settings, such as those by Khan et al. (24), Spasojevic et al. (27), and Yeung et al. (31), benefited from controlled environments and professional staff oversight. In contrast, home-based deployments like the BESI study by Bankole et al. (30) and the work by Bafaloukou et al. (33) faced greater variability in environmental conditions but offered higher ecological validity by capturing agitation in the patient’s natural living environment. Bankole et al. (30) explicitly noted a preference for home-based monitoring systems among PwD and caregivers, highlighting the importance of developing systems that blend into daily life outside of clinical facilities.

## 4 Discussion

One of the primary aims of this review was to explore two central hypotheses: first, that wearable sensor systems may be capable of detecting early signs of agitation in PwD; and second, that personalized ML models can offer improved accuracy in agitation detection compared with generic approaches. Although the current evidence base remains emergent and somewhat preliminary, findings suggest promising support for both hypotheses. Wearable sensors, often combined with environmental and multimodal monitoring systems, demonstrate the capacity to identify behavioral symptoms such as agitation and aggression in dementia patients with reasonable accuracy. Furthermore, tailoring ML algorithms to individual patient characteristics appears to enhance detection performance, highlighting the potential benefits of personalized, patient-centered approaches. As highlighted in many studies explored in this review, environmental and contextual influences, such as caregiver interactions and sensory stimuli, can play a significant role in impacting agitation. By incorporating these factors into early detection methods, caregivers may better understand and anticipate agitation episodes. This improved understanding could support more effective responses, enhance caregiver-patient dynamics, and potentially contribute to the well-being of both patients and caregivers, fostering a positive feedback loop that may lead to better management and care outcomes. Nevertheless, more rigorous, long-term research is necessary to substantiate these conclusions and translate them into clinical practice.

To contextualize the evidence base underlying these conclusions, the included studies differed not only in sensor configurations but also in analytic strategies, ranging from feasibility and association-based analyses to trained predictive models. Across the included studies summarized in Supplementary File S2, seven studies implemented ML or DL approaches, whereas four relied primarily on non-ML analytic approaches. Within this literature, generic approaches include population-level (non-personalized) models applied uniformly across participants without subject-specific calibration, as well as non-ML approaches focused on feasibility, descriptive monitoring, or associations with observation-based measures rather than predictive classification. Only a limited subset of studies explicitly described or evaluated personalization; where reported, individualized modeling was described as outperforming generic baselines. Reported ML/DL performance was task- and metric-dependent, with some studies reporting task-specific accuracy (e.g., 93.45%–98.6%) and others reporting discrimination or sensitivity/specificity-based performance. The ML/DL methods represented across studies included acoustic/speech-based event detection, multimodal feature-based classification, LSTM-based forecasting using environmental cues, interpretable boosting models (LightGBM), video-based spatiotemporal DL via convolutional autoencoder architectures, and neural-network–based signal-quality classification. These methodological trends are consistent with the broader rationale for digital biomarkers, namely that physiological and behavioral signatures captured by sensors may require computational models that can integrate inter-individual variability and contextual complexity.

Current wearable sensor technologies may offer a unique opportunity to capture physiological and behavioral signals that may reflect some of the complex neurobiological processes underlying agitation, such as dysregulation in serotonergic, noradrenergic, and GABAergic systems, as well as disruptions in cortical–limbic circuitry. Digital biomarkers including heart rate variability, electrodermal activity, respiratory patterns and motor activity, can serve as accessible proxies for these neurochemical and neural disturbances. By leveraging personalized ML models to analyze these multimodal signals, it becomes possible to approximate an individual’s unique neurobiological profile, facilitating earlier detection and more tailored interventions. While these technological and analytical approaches remain in development, their integration with neurobiological insights presents a promising avenue for advancing the assessment and management of agitation in dementia care.

These results underline the importance of user-centered design principles in the development of sensor technologies for dementia care. Challenges in the way of adherence with wearable sensors have highlighted the need for solutions that are technologically advanced yet user-friendly and comfortable for the dementia- afflicted. The prospect of individually tailored monitoring systems capable of adapting to the distinct needs and behaviors of each patient opens entirely new perspectives for customizing interventions and increasing the relevance of monitoring data. Also, the application of artificial intelligence techniques is very promising in improving the prediction and detection of behavioral symptoms through the detailed analysis of sensor data and environmental indicators.

Looking ahead to future research in this area, it is posited that extensive longitudinal studies on diverse populations may further the understanding of the long-term impacts sensor-based monitoring has on the well-being of PwD. More validity studies in real clinical settings would further increase the reliability and believability of sensor technologies, thus easing their wider adoption into dementia care practices. Future research should prioritize larger, prospective validation studies enrolling adequate samples with independent test sets across multiple sites, explicitly documenting dementia type, disease stage, presence/absence of delirium, and comorbidities to improve generalizability. Additionally, the adoption of standardized, validated agitation assessment tools and delirium screening protocols would enable more consistent and comparable study outcomes. Long-term longitudinal studies are essential to track not only technical performance but also clinical outcomes such as medication use, behavioral incidents, quality of life, caregiver burden, and system adherence. Intentional recruitment of diverse could also help ensure technologies are equitable and generalizable across different groups.

Another important consideration that would be helpful for future projects in the field is the fact that any wearable technologies used to gather information from patients would need to meet relevant standards developed by the International Organization for Standardization (ISO). Mainly, conformance to such standards would be for the assurance of safety, quality, and effectiveness. According to the literature, some important ISO standards are especially relevant in the development and deployment of technologies like those highlighted in our review. ISO 14971, for example, focuses on risk management throughout a device’s life cycle in order to avoid failures that could endanger users. ISO 13485 outlines the requirement for a quality management system that assists in ensuring medical devices meet regulatory requirements for their manufacture. The ISO 11073 series promotes effective communication and data exchange among medical devices, hence improving interoperability. ISO 10993 assures that devices in contact with human tissue are biocompatible, whereas ISO 60601 focuses on the safety and performance of medical electrical equipment. Furthermore, ISO 9241 encourages usability, while ISO/IEC 25010 defines key software quality features, both of which contribute to the dependability and user experience of wearable technology in healthcare. Therefore, it is advisable that any devices used be thoughtfully designed to comply with as many relevant ISO standards as possible (35, 36).

One important aspect often overlooked in our effort to understand agitation in dementia care is the person’s unique, unmet needs. Looking to the future, it will become all the more imperative that we tease out the tangled connections between episodes of agitation and these specific needs. Filling this knowledge gap, future research has the potential to empower care provision in such a way that it can more thoroughly target interventions to address basic human needs in people experiencing dementia. It is important to underline that the core of these interventions lies in their being people-centered. The purpose should always be to simplify rather than complicate the lives of those we are trying to help, so as to improve their well-being and quality of life.

Future studies should move beyond detection-only systems to develop integrated platforms combining sensor-based detection, decision-support tools, and personalized intervention strategies that are actionable for caregivers and clinicians. Ultimately, randomized controlled trials testing whether sensor-based detection combined with personalized interventions improves clinical outcomes compared to standard care are essential.

Lastly, expanding the research scope beyond dementia to other conditions where communication and expression of needs are compromised may provide valuable insights into the wider applicability of sensor- based monitoring technologies. Examining the potential for these technologies to support individuals with conditions such as autism spectrum disorder or traumatic brain injury, many new approaches toward better communication and improved quality of life for a wide variety of people with special care needs could be realized.

Although this study provides valuable insights into the potential of sensor-based monitoring systems for detecting agitation in PwD, several limitations must be acknowledged. As with all scoping reviews, there is always a possibility that some relevant literature may have been overlooked or that methodological flaws in included studies could introduce certain biases into the outcomes. Notably, the generalizability of current findings is often constrained by small sample sizes and a lack of prospective validation on independent test sets or in diverse care settings. Nevertheless, we believe this review marks a significant step forward in understanding the applications of wearable and environmental technologies in dementia care and identifying future research directions.

The short chronology of widespread adoption of these technologies complicates the ability to make definitive conclusions: much of the existing literature consists of cross-sectional data or preliminary studies, which might not fully represent long-term dependability, effectiveness, or broader implications of sensor-based systems on patient outcomes. Additionally, the limited longitudinal follow-up in most studies restricts understanding of how system performance evolves over time, how detection patterns change with disease progression, and whether interventions informed by these systems impact long-term outcomes such as quality of life or caregiver burden.

Added to this is the complexity of agitation in dementia, influenced by both psychosocial and pathophysiological factors, which provides significant challenges to determining definitive and clear- cut endpoints. Thus, heterogeneity in how agitation was defined and measured across studies also constrains comparability and makes it difficult to determine true algorithm performance. A further limitation arises from the lack of studies that fully explore the psychological impact of using these technologies in PwD. More specifically, we felt that there was a need for more studies that actively involve patients themselves and elicit information about how these interventions have impacted their quality of life and psychological well-being. Moreover, the aforementioned complexities are often compounded by the inadequate assessment of confounding factors, as few studies systematically controlled for delirium, medical comorbidities, or medication effects that can substantially alter sensor signals. Finally, we believe that this patient-centered focus should also extend to rigorous ethical analysis, addressing largely overlooked issues such as informed consent in cognitively impaired populations, data privacy, and the potential for algorithmic bias. This review recognizes that the field of sensor-based monitoring in dementia care is still in its development. The aim of this work is not to provide a set of definitive conclusions but to contribute to the growing body of literature and identify pragmatic steps to improve patient outcomes and caregiver experiences. Future research should address these shortcomings by conducting in-depth longitudinal studies, progressing detection algorithms, and developing protocols for the safe, effective integration of these technologies into clinical practices.

## 5 Conclusion

In the last decade, the integration of wearable sensors has shown great potential in real-time detection and management of behavioral and psychological symptoms. Within this framework, our literature review aimed to explore the potential of wearable technology applied to health care, especially for people with neurodegenerative diseases who face big challenges in communication and agitation. The results show that these technologies can be useful in increasing remote monitoring and defining early detection, thereby having the potential to empower both patients and their caregivers.

Personalized ML models have demonstrated the potential to improve symptom classification accuracy and facilitate timely, tailored interventions, thus enhancing overall patient care. The concept of digital phenotyping - a method of leveraging wearable sensor data to quantify individual human phenotypes - arises as very promising in elucidating and managing such complex conditions. Moreover, the combination of state-of-the-art artificial intelligence methods has been effective at improving the predictive capabilities of such systems, enabling anticipatory and individualized care. Most of the studies we reviewed favor a multimodal approach, since there are a number of studies that report outcomes suggesting it is a way ahead in the future with upcoming developments in the area of healthcare. Finally, our review highlights the importance of user-centered design principles in the development of wearable technologies. For these devices to achieve high compliance and effectiveness in real-world settings, it is of utmost importance that they are comfortable, private, and user-friendly.

Our recommendations for future research include large-scale longitudinal studies, as well as validation in diverse clinical settings, to find out more about the long-term efficacy and reliability of these technologies. Furthermore, we believe that expanding the use of wearable sensors in other conditions characterized by impairments in communication, such as autism spectrum disorder and traumatic brain injury, may enable deeper insight into the causes of some of the behavioral manifestations and their subsequent consequences. In conclusion, we support the idea that human-centered design - addressing the needs of each particular patient—is one of the best strategies for providing truly effective and caring health interventions.

## Data Availability

The original contributions presented in the study are included in the article/Supplementary material, further inquiries can be directed to the corresponding author.
